# Effects of a combination treatment of KD5040 and _L_-dopa in a mouse model of Parkinson’s disease

**DOI:** 10.1186/s12906-017-1731-2

**Published:** 2017-04-19

**Authors:** Sora Ahn, Taek-Jin Song, Seong-Uk Park, Songhee Jeon, Jongpil Kim, Joo-Young Oh, Jaehwan Jang, Sanhwa Hong, Min-A Song, Hye-Seoung Shin, Young-Rim Jung, Hi-Joon Park

**Affiliations:** 1 Integrative Parkinson’s Disease Research Group, Acupuncture & Meridian Science Research Center, Kyung Hee University, 26 Kyungheedae-ro, Dongdaemoon-gu, Seoul, 02447 Republic of Korea; 2 Acupuncture and Meridian Science Research Centre (AMSRC), Kyung Hee University, 26 Kyungheedae-ro, Dongdaemun-gu, Seoul, 02447 Republic of Korea; 3 Graduate School of Korean Medicine, Kyung Hee University, 26 Kyungheedae-ro, Dongdaemun-gu, Seoul, 02447 Republic of Korea; 4 Stroke and Neurological Disorders Center, Kyung Hee University Hospital at Gangdong, Seoul, 05278 Republic of Korea; 5 Department of Biomedical Engineering, Dongguk University, Sangyoung-Bio, Biomedi-Campus, Dongguk-ro 32, Goyang-si, Gyeonggi-do 10326 Republic of Korea; 6 Dongguk University Research Institute of Bio-Medi Integration, Sangyoung-Bio, Biomedi-Campus, Dongguk-ro 32, Goyang-si, Gyeonggi-do 10326 Republic of Korea; 7 SL. BIOTECH, Gasan Digital 1-ro 189, Geumcheon-gu, Seoul, 08592 Republic of Korea; 8 Seoul Pharma Laboratory, Gasan Digital 2-ro 14, Geumcheon-gu, Seoul, 08592 Republic of Korea

**Keywords:** KD5040, _L_-dopa combination treatment, Parkinson’s disease, _L_-dopa-induced dyskinesia, Synergic effect, Enkephalin, Substance P, FosB

## Abstract

**Background:**

Although the dopamine precursor L-3, 4-dihydroxyphenylalanine (_l_-dopa) remains the gold standard pharmacological therapy for patients with Parkinson’s disease (PD), long-term treatment with this drug has been known to result in several adverse effects, including _l_-dopa-induced dyskinesia (LID). Recently, our group reported that KD5040, a modified herbal remedy, had neuroprotective effects in both in vitro and in vivo models of PD. Thus, the present study investigated whether KD5040 would have synergistic effects with _l_-dopa and antidyskinetic effects caused by _l_-dopa as well.

**Methods:**

The effects of KD5040 and _l_-dopa on motor function, expression levels of substance P (SP) and enkephalin (ENK) in the basal ganglia, and glutamate content in the motor cortex were assessed using behavioral assays, immunohistochemistry, Western blot analyses, and liquid chromatography tandem mass spectrometry in a mouse model of PD induced by 1-methyl-4-phenyl-1,2,3,6-tetrahydropyridine (MPTP). In addition, the antidyskinetic effects of KD5040 on pathological movements triggered by _l_-dopa were investigated by testing abnormal involuntary movements (AIMs) and measuring the activations of FosB, cAMP-dependent phosphor protein of 32 kDa (DARPP-32), extracellular signal-regulated kinases (ERK), and cAMP response element-binding (CREB) protein in the striatum.

**Results:**

KD5040 synergistically improved the motor function when low-dose _l_-dopa (LL) was co-administered. In addition, it significantly reversed MPTP-induced lowering of SP, improved ENK levels in the basal ganglia, and ameliorated abnormal reduction in glutamate content in the motor cortex. Furthermore, KD5040 significantly lowered AIMs and controlled abnormal levels of striatal FosB, pDARPP-32, pERK, and pCREB induced by high-dose _l_-dopa.

**Conclusions:**

KD5040 lowered the effective dose of _l_-dopa and alleviated LID. These findings suggest that KD5040 may be used as an adjunct therapy to enhance the efficacy of _l_-dopa and alleviate its adverse effects in patients with PD.

## Background

Parkinson’s disease (PD) is the second most common neurodegenerative disorder. It manifests as the deterioration of dopaminergic neurons in the substantia nigra pars compacta (SNpc) and the subsequent depletion of dopamine in the striatum [1], leading to the well-known motor symptoms associated with PD, including akinesia, resting tremor, bradykinesia, and rigidity [2, 3].

The current treatment approaches for PD are dopamine-centric and typically include L-3,4-dihydroxyphenylalanine (_L_-dopa), which is a dopamine precursor and/or dopaminergic agonist. Although these approaches have been successful, alternative treatment strategies need to be developed because the long-term use of _L_-dopa demands progressive dose escalations and inevitably results in various complications, including motor fluctuations referred as _L_-dopa-induced dyskinesia (LID) [4]. In addition, _L_-dopa has little effect on several non-motor PD symptoms such as dysautonomia, sleep disturbances, cognitive impairments, and apathy [5]. A number of non-dopaminergic treatments have been suggested for PD due to its diversity of symptoms but have produced limited clinical benefits.

To identify a novel adjunct therapy with potential synergistic effects in combination with _L_-dopa many herbal formulas were evaluated; ultimately, a modified form of Cheong-Gan-Tang (CGT) was identified as the most promising candidate. CGT is used in traditional East Asian medicine for the treatment of motor-related disorders, such as PD. It consists of six crude herbs: *Paeonia lactiflora* Pall, *Ligusticum chuanxiong* Hort, *Angelica gigas* Nakai, *Bupleurum falcatum* Linne, *Gardenia jasminoides* Ellis, and *Paeonia suffruticosa* Andrews. To improve the treatment efficacy of CGT, a modified formula named KD5040, consisting of CGT plus *Eugenia caryophyllata* Thunb (ECT) and *Pogostemon cablin* Bentham (PCB) with a high degree of free radical-scavenging activity were developed [6]. In a previous study by our group, KD5040 showed neuroprotective effects and inhibited 6-hydroxydopamine (6-OHDA)-induced c-Jun N-terminal protein kinase (JNK) phosphorylation and apoptosis in primary dopamine neurons in a PD-like phenotype [6]. In addition, an in vivo study that used a mouse model of PD induced by 1-methyl-4-phenyl-1,2,3,6-tetrahydropyridine (MPTP) demonstrated that KD5040 improves motor function, rescues dopaminergic neurons, and improves the expression level of tropomyosin receptor kinase A (TrkA), which is involved in neuronal differentiation [6].

To widen the therapeutic window of KD5040, the present study investigated whether the co-administration of KD5040 and _L_-dopa would improve motor function and alleviate LID compared to _L_-dopa alone. The anti-parkinsonian effects of KD5040 were evaluated using behavioral tests and by measuring the expression levels of substance P (SP) and enkephalin (ENK), which regulate the dopaminergic pathways in PD. In addition, the antidyskinetic effects of KD5040 on pathological movements induced by _L_-dopa were investigated by testing abnormal involuntary movements (AIMs) and measuring the activations of FosB, cAMP-dependent phosphor protein of 32 kDa (DARPP-32), extracellular signal-regulated kinases (ERK), and cAMP response element-binding (CREB) protein in the striatum.

## Methods

### Animals

Male C57BL/6 mice (Central Lab Animal, Inc., Seoul, Republic of Korea), 9 weeks of age and weighing 21–25 g were used. Animals were maintained on a 12/12-h light/dark cycle at a constant room temperature of 24 ± 1 °C. All experiments were approved by the Kyung Hee University Animal Care Committee for animal welfare (KHUASP(SE)-14–052) and were performed according to the guidelines of the National Institutes of Health and the Korean Academy of Medical Sciences.

### Preparation of KD5040 and control of standard biomarkers

To prepare an extract of CGT, 100 g of a mixture containing rhizome of *Paeonia lactiflora* Pall (Paeoniaceae, 29.41 g), rhizome of *Ligusticum chuanxiong* Hort (Umbelliferae, 19.61 g), rhizome of *Angelica gigas* Nakai (Umbelliferae, 19.61 g), rhizome of *Bupleurum falcatum* Linne (Umbelliferae, 15.69 g), fructus of *Gardenia jasminoides J.* Ellis (Rubiaceae, 7.84 g), and root peel of *Paeonia suffruticosa* Andrews (Paeoniaceae, 7.84 g), was pulverized and extracted twice with 10 vol. of 30% ethanol at 100 °C with a reflux condenser for 3 h, then filtered with a 50 μm filter and lyophilized with a freeze dryer. The final yield from the whole procedure was 15.47 g of dried mixture (average yield =15.47%). To increase the effect of CGT, other herbs were extracted. The yield of flower buds of *Eugenia caryophyllata* Thunb (ECT) (Myrtaceae, 100 g) was 10.69 g (average yield =10.69%), and the top part of *Pogostemon cablin* Bentham (PCB) (Labiatae, 100 g) was ~6 g (average yield = ~6%). Herbs were provided by the Department of Pharmacy of Oriental Medicine, Dongguk Medical Center, for research use. The quality of each herb was identified and authenticated by Prof. Byung-Soo Koo (Korean Medical Hospital, Dongguk University). A sample of KD5040 was deposited at the Kyung Hee University herbarium (deposit #: KHH-G-0054). To obtain KD5040, CGT was mixed with ECT and PCB at a ratio of 3:1:1. The dried material was stored at −80 °C until use.

### Experimental design

To develop a reliable PD model, 1-methyl-4-phenyl-1, 2, 3, 6-tetrahydropyridine (MPTP, 30 mg/kg, Sigma, St Louis, MO, USA) or saline (MPTP vehicle) was injected intraperitoneally for 5 consecutive days. Then, 1 week after the last MPTP injection, each dose of _l_-dopa (5 and 10 mg/kg to test synergistic effects with KD5040 and 20 mg/kg to test the anti-dyskinetic action of KD5040) or saline (_l_-dopa vehicle) was injected intraperitoneally to the randomly assigned mice for 8 days. Mice were fed an experimental diet supplement with or without KD5040 for 8 days from 1 week after the last MPTP injection.

**Fig. 1:**
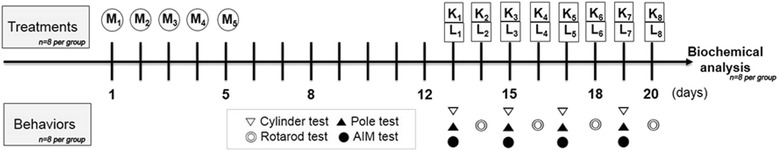
Schematic representation of the experimental design. Each mouse received an injection of either saline or MPTP solution for 5 consecutive days. One week after the final injection, the mice were administered _l_-dopa (5, 10, and 20 mg/kg) along with KD5040 daily for 8 days. M: MPTP, K: KD5040, L: _l_-dopa

To test the synergistic effects of KD5040 and _l_-dopa and the anti-dyskinetic action of KD5040, mice were divided randomly into eight groups (each *n* = 8 per group). Control group mice received the MPTP vehicle (saline for 5 days, i.p.) and the _l_-dopa vehicle (saline, i.p.). MPTP group mice received MPTP (30 mg/kg/day for 5 days, i.p.) and _l_-dopa vehicle (saline, i.p.). LL group mice received MPTP + a low dose of _l_-dopa (5 mg/kg, i.p.). LL + KD group mice received MPTP + a low dose of _l_-dopa (5 mg/kg, i.p.) + KD5040. ML group mice received MPTP + a medium dose of _l_-dopa (10 mg/kg, i.p.). ML + KD group mice received MPTP + a medium dose of _l_-dopa (10 mg/kg, i.p.) + KD5040. HL group mice received MPTP + a high dose of _l_-dopa (20 mg/kg). HL + KD group mice received MPTP + a high dose of _l_-dopa (20 mg/kg) + KD5040. The detailed experimental schedule is shown in Fig. 1.

### Behavioral tests

Behavioral tests were performed to measure the anti-dyskinetic effects of KD5040 as well as the synergistic effects of the combination treatment of _l_-dopa and KD5040. Behavioral tests were started 1 week after the last MPTP injection. All tests were performed 20 min after _l_-dopa injection. All experiments were performed in an assessor-blinded manner to decrease the risk of bias. To test the synergistic effects of _l_-dopa and KD5040, rotarod, pole, and cylinder tests were used. Additionally, modified abnormal involuntary movement (AIM) tests were used to assess the anti-dyskinetic actions of KD5040. The detailed schedule is shown in Fig. 1.

#### Rotarod test

The rotarod test was used to evaluate neurological impairment, such as fore and hind limb motor coordination and balance [7]. The rod instrument (MED Associates, Inc., VT, USA) was used to record the falling time from the rod. The time on the rod was recorded with a maximum 480 s at successive rod speeds (0 to 35 rpm).

#### Pole test

The pole test was to measure bradykinesia in the PD model [8]. The instrument consists of a 55 cm-high pole and 1.3 cm in diameter. The mouse was placed head upward near the top of the pole, and the time taken for the mouse to reach the floor was determined.

#### Cylinder test

The mice were placed in a plastic cylinder (12 cm in diameter and 20 cm tall) for 5 min without habituation. Observers who were blinded to the experiments counted the number of forelimb wall contacts.

#### AIM test

We used a modified AIM assessment to evaluate LID in the mice [9, 10]. AIMs induced by _l_-dopa were measured 20 min after _l_-dopa (20 mg/kg, i.p.) administration. The middle 3 min of the video recording of the 5-min cylinder test were analyzed. Scores were the sum of the following: (1) front-paw dyskinesia: repetitive rhythmical spasm, or dystonic posture of the front paws; (2) three-paw dyskinesia: both front-paw dyskinesia with one of the hind paws moving up and down; and (3) jumping dyskinesia: rapid jumping in the cylinder.

### Brain tissue preparation and immunohistochemistry

At 20 days after the first MPTP injection, mice were sacrificed and perfused transcardially with cold 4% paraformaldehyde (PFA) in 0.2 M phosphate buffer. The brains were removed, post-fixed in 4% PFA overnight at 4 °C, and then soaked in 30% sucrose and processed for cryoprotection. The frozen brains were cut into 40-μm coronal sections using a freezing microtome (CM1850; Leica, Germany) and stored in cryoprotectant (30% ethylene glycol, 30% glycerol, and 0.02 M PB) at 4 °C until use. Next, brain sections were washed in phosphate buffered saline (PBS) and treated with 3% H_2_O_2_ in 0.05 M PBS, and the sections were blocked with 1% bovine serum albumin and normal goat serum. Then, they were incubated with rabbit anti-met-enkephalin (1:1000, Immunostar, Inc., WI, USA), anti-substance P (1:1000, Immunostar, Inc., WI, USA), or rabbit anti-FosB (1:500, Cell Signaling Technology, MA, USA) overnight at room temperature. Then, sections were incubated with biotinylated anti-rabbit IgG (Vector Laboratories, Inc., CA, USA) for 1 h, followed by avidin–biotinylated peroxidase complex (Vectastain Elite ABC kit; Vector Laboratories, Inc., CA, USA) and diaminobenzidine (Sigma, St. Louis, MO, USA) as the developing agent. Sections were mounted on gelatin-coated slides, dried, dehydrated, and coverslipped. The Histological images were examined using a bright-field microscope (BX51; Olympus Japan Co., Tokyo, Japan). All procedures for analyses were performed in a blinded manner to reduce the risk of observer bias.

### Western blot analysis

Brain tissues were detached by scraping and sonicated for 90 s in lysis buffer. Sample was mixed loading buffer (2% SDS, 5% 2-mercaptoethanol, 25% glycerol, and 0.2 mg/ml bromphenol blue in 125 mM Tris-HCl, pH 6.8), heated at 95 °C for 5 min, and separated by 16% Tris-Tricine sodium dodecyl sulfate polyacrylamide gel electrophoresis (SDS-PAGE). Proteins were separated by 10% SDS-PAGE and then transferred to an Immobilon-P membrane (Millipore, Bedford, MA). The blotted membrane was blocked with 5% skim milk in PBS containing 0.05% Tween 20 (PBS-T buffer) for 1 h. After washing the membrane with PBS-T, each antibody diluted in PBS-T containing 0.25% BSA, was added and incubated for overnight at 4 °C. Blots were incubated with antibodies against enkephalin (1:1000, Immunostar, Inc., WI, USA), substance P (1:1000, Immunostar, Inc., WI, USA), FosB (1:500, Cell Signaling Technology, MA, USA), pCREB (1:200, Cell Signaling Technology, MA, USA), pDARRP32 (1:200, Cell Signaling Technology, MA, USA), and β-actin (1:5000, Sigma, St Louis, MO, USA). After washing with Tris-buffered saline containing 0.05% Tween 20, the blots were incubated with horseradish peroxidase-conjugated secondary rabbit (catalog PA1–30359, Thermo Scientific, USA) and mouse (catalog PA1–30355, Thermo Scientific, MA, USA) antibodies. Detection was performed by using enhanced chemiluminescence (ECL kit, Thermo Fisher Scientific, MA, USA) and images were obtained using Molecular Imager ChemiDoc XRS+ (Bio-Rad, CA, USA). Band intensity was analyzed with the Image Lab software (ver. 2.0.1; Bio-Rad, CA, USA).

### Measurements of glutamate content in the motor cortex by liquid chromatography tandem mass spectrometry (LC-MS/MS)

We analyzed the levels of glutamate in the motor cortex by LC-MS/MS [11]. The brain tissue (motor cortex) was prepared by diluting it with 0.1% formic acid in 5 mL methanol solution. The tubes were shaken vigorously on a multi-tube vortexer (VWR, USA) for 30 min, followed by centrifugation (3000 rpm, 5 min). The supernatant (1 mL) was put on Strata Impact protein precipitation 2-mL square well filter plates and allowed to stand for 5 min to obtain a sample drop-down rate of 2 drops/s. Part (5 μL) of each sample was injected for analysis by LC-MS/MS. The LC-MS/MS system consisted of a UFLC XR system (Shimadzu, Japan) and a QTRAP5500 (ABSciex, USA), and analytical separation was achieved using a Capcellpak C18 MG (2.0 × 150 mm, 5 μm, Shiseido, Japan). The mobile phase, consisting of 2.5 mM NFPA in water and 2.5 mM NFPA in acetonitrile (85:15; *v*/v), was delivered at a flow rate of 0.2 mL/min; total run time was 10 min. The linear calibration curve of _l_glutamic acid was estimated using the peak area ratio of the analyte in high-performance liquid chromatography (HPLC), using a weighting factor of 1/X^2^ (where X = peak area ratio). Parameters obtained from the calibration curve were used to determine the concentration of the unknown samples by back-calculation.

### Gas chromatography-mass spectrometry (GC-MS) analysis of compounds of KD5040

To analyze the compounds in KD5040, we performed GC, equipped with an Rtx-5 ms column (30 m × 0.25 mm × 0.25 μm) and a mass spectrometer (GCMS-QP5000 series; Shimadzu, Japan). The carrier gas was hydrogen at a flow rate of 3 mL/min. The column temperature was initially at 40 °C for 1 min, then increased gradually to 300 °C at 10 °C/min. Extracts were diluted 1:10 (*v*/v) with ethanol, and 1 μL of the diluted samples was injected automatically in split mode. Injector and detector temperatures were set at 230 °C and 280 °C, respectively.

### Statistical analysis

GraphPad Prism (ver. 5; GraphPad Software, Inc., San Diego, CA, USA) was used for statistical analyses. Results other than AIMs were analyzed using one-way ANOVA. AIMs data were analyzed using two-way ANOVA, considering group and time. All results are expressed as means ± SEMs. *P*-values <0.05 were considered to indicate statistically significant results.

## Results

### Behavioral assessments of the synergistic effects of _l_-dopa and KD5040

**Fig. 2:**
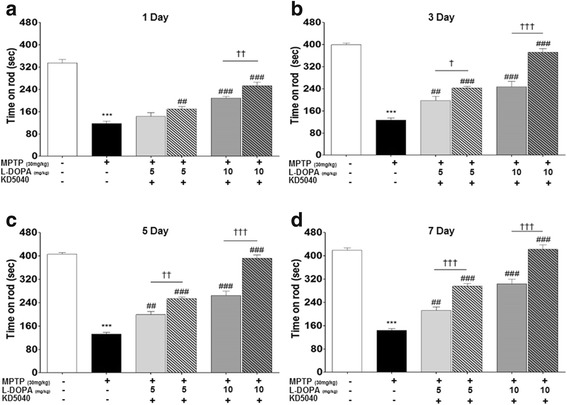
Effects of combination treatment on days 2 (**a**), 4 (**b**), 6 (**c**), and 8 (**d**) after first treatment with KD5040 based on the rotarod test. The _l_-dopa groups (LL and ML) exhibited dose-dependent increases in latency time, and the combination treatment resulted in synergistic increases. C: control, M: MPTP, K: KD5040, LL: low dose of _l_-dopa (5 mg/kg), ML: medium dose of _l_-dopa (10 mg/kg). *** *p* < 0.001 compared to control, ### *p* < 0.001 and ## *p* < 0.01 compared to MPTP, and ††† *p* < 0.001, †† *p* < 0.01, and † *p* < 0.05 compared to each dose of _l_-dopa

**Fig. 3:**
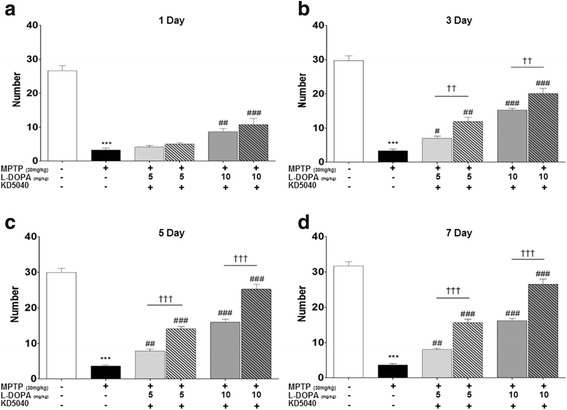
Effects of combination treatment on days 1 (**a**), 3 (**b**), 5 (**c**), and 7 (**d**) after first treatment with KD5040 based on the rearing test. The number of rearing behaviors in both _l_-dopa groups (LL and ML) significantly increased relative to the MPTP group, which increased further after co-administration with KD5040. C: control, M: MPTP, K: KD5040, LL: low dose of _l_-dopa (5 mg/kg), ML: medium dose of _l_-dopa (10 mg/kg). ***p < 0.001 compared to control, ### *p* < 0.001 and ##*p* < 0.01 compared to MPTP, and ††† *p* < 0.001 and †† *p* < 0.01, compared to each dose of _l_-dopa

**Fig. 4:**
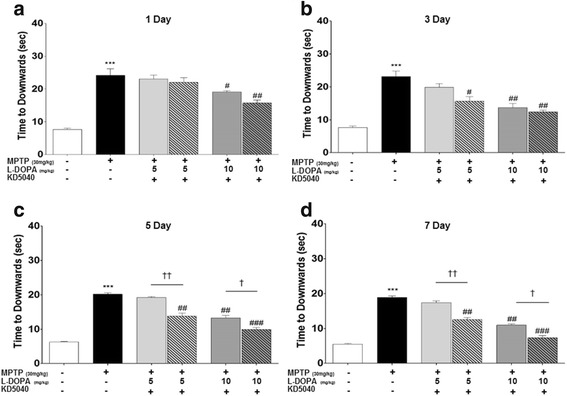
Effects of combination treatment on days 1 (**a**), 3 (**b**), 5 (**c**), and 7 (**d**) after first treatment with KD5040 based on the pole test. KD5040 in combination with _l_-dopa (LL and ML) reduced time to descend compared to the respective doses of _l_-dopa alone. C: control, M: MPTP, K: KD5040, LL: low dose of _l_-dopa (5 mg/kg), ML: medium dose of _l_-dopa (10 mg/kg). *** *p* < 0.001, ** *p* < 0.01, and * *p* < 0.05 vs. control, ### *p* < 0.001, ## *p* < 0.01, and # *p* < 0.05 vs. MPTP, and ††† *p* < 0.001, †† *p* < 0.01, and † *p* < 0.05 vs. each dose of _l_-dopa

Muscle strength and ability to balance gradually decreased in the MPTP group. In addition, motor function tests revealed that mice treated with MPTP exhibited significantly lower performance from days 13 to 20 after the first MPTP injection compared to the control group (Figs. 2, 3 and 4). These findings confirm that our PD model was well developed and sustained.

An analysis of latency times in the rotarod test revealed dose-dependent increases in latency in the _l_-dopa groups (LL and ML) compared to the MPTP group (*p* < 0.01 and 0.001 in LL and ML, respectively, vs. MPTP) on day 7 after initiation of _l_-dopa treatment. Furthermore, on day 7 after treatment, KD5040 and _l_-dopa produced synergistic increases in latency (*p* < 0.001 vs. both LL and ML) that first appeared on day 3 after treatment. Overall, combination treatment with KD5040 and 5 mg/kg _l_-dopa (which is half the standard dose) produced the same degree of improvement in the rotarod test compared to a higher dose of _l_-dopa (10 mg/kg) alone (Fig. 2).

Behavioral results of the cylinder wall touch test were similar to those of the rotarod test. The number of cylinder wall touches in both _l_-dopa groups (LL and ML) showed a dose-dependent increase compared to the MPTP group (*p* < 0.01 and 0.001 in LL and ML, respectively, vs. MPTP) on day 7 of treatment. Furthermore, combination treatment with KD-5040 and _L_-dopa produced significantly higher cylinder wall touches on day 7 (each *p* value <0.001 vs. LL and ML; Fig. 3).

The results of pole tests also confirmed the behavioral benefits of combination treatment with _l_-dopa and KD5040. The group receiving a medium dose of _l_-dopa (ML) showed lower time to descend compared to the MPTP group. Similarly, the groups receiving KD5040 and _l_-dopa (5 and 10 mg/kg, LL and ML, respectively) exhibited lower time to descend compared to the individual doses of only _l_-dopa (*p* < 0.01 and 0.05, respectively) and the MPTP group (*p* < 0.01 and 0.001, respectively; Fig. 4).

### Effects of combination treatment on expression levels of ENK and SP

**Fig. 5:**
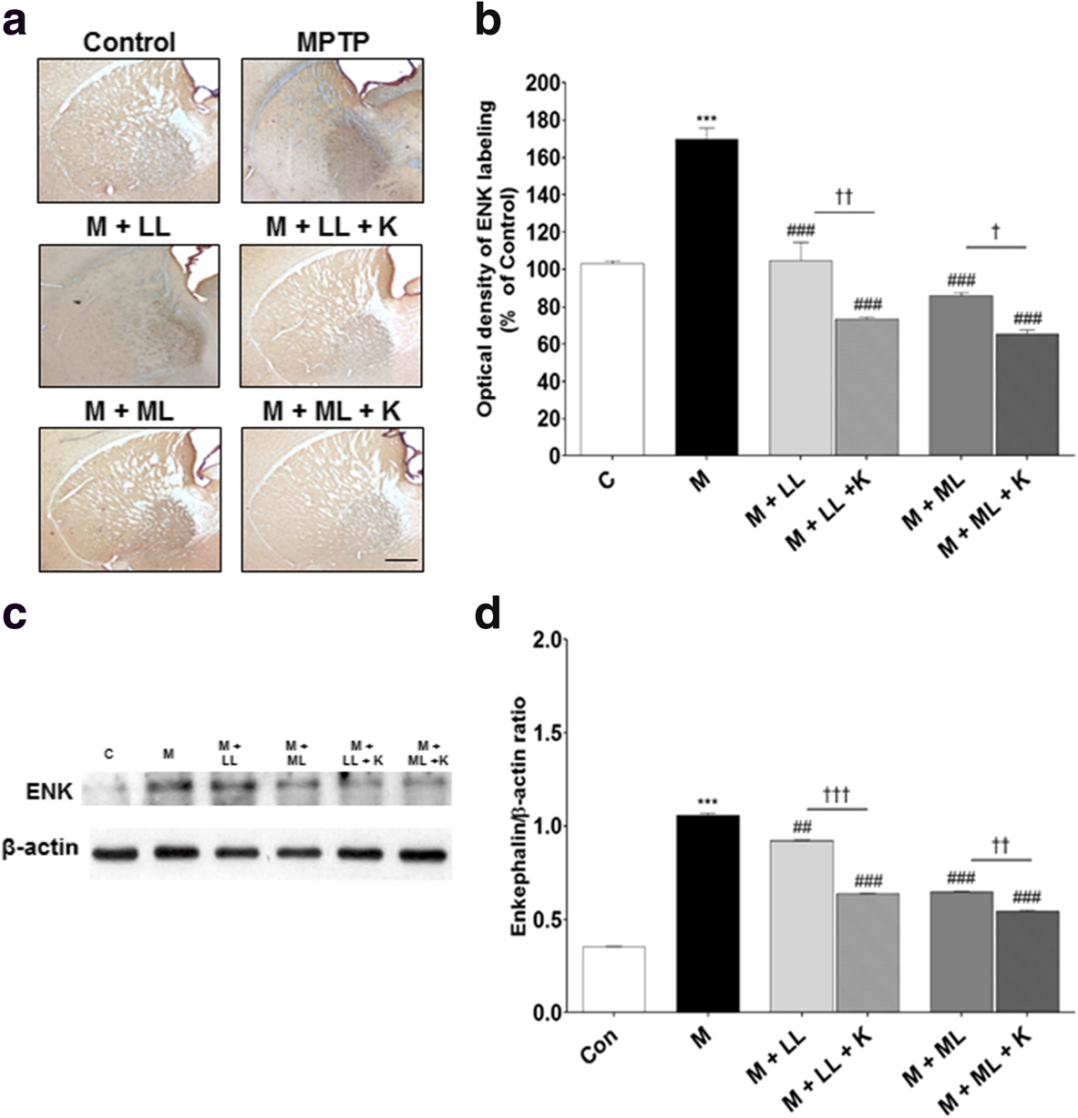
Effects of combination treatment on ENK expression in the GPe. Immunohistochemical analyses of ENK expression in the GP (**a**, **b**). Western blot analyses of ENK expression in the GP (**c**, **d**). The MPTP group showed higher ENK immunoreactivity in the GPe, but combination treatment reduced ENK expression in the GP. C: control, M: MPTP, K: KD5040, LL: low dose of _l_-dopa (5 mg/kg), ML: medium dose of _l_-dopa (10 mg/kg). *** *p* < 0.001 vs. control, ### *p* < 0.001 vs. MPTP, and †† *p* < 0.01 vs. each dose of _L_-dopa. Error bars represent SEM. Scale bar: 200 μm. ENK: enkephaline; GPe: external segment of globus pallidus

To determine the behavioral benefits of combination treatment with _l_-dopa and KD5040, the expression levels of ENK in the external segment of the globus pallidus (GPe) and of SP in the ventral midbrain area were assessed via immunohistochemistry. The MPTP group exhibited higher ENK immunoreactivity in the GPe (169.9 ± 11.9% vs. control), but _l_-dopa treatment (LL and ML) attenuated such increases (LL: 109.9 ± 7.5%; ML: 86.1 ± 3.5%; both *p* values <0.001 vs. MPTP). Combination treatment with KD5040 and _l_-dopa augmented the decreased expression of ENK in both _l_-dopa groups (*p* < 0.01 vs. LL and <0.05 vs. ML; Fig. 5a and b). Western blot analyses confirmed higher ENK expression in the MPTP group (298.6 ± 0.1%, *p* < 0.001 vs. control), whereas ENK expression was clearly inhibited in the LL (259.9 ± 0.1%, *p* < 0.01) and ML (183.1 ± 0.1%, *p* < 0.001) groups. Furthermore, combination treatment with KD5040 accelerated this decrease (with LL: 179.6 ± 0.1%; with ML: 153.5 ± 0.2%; both *p* values <0.001 vs. each dose of _l_-dopa group).

**Fig. 6:**
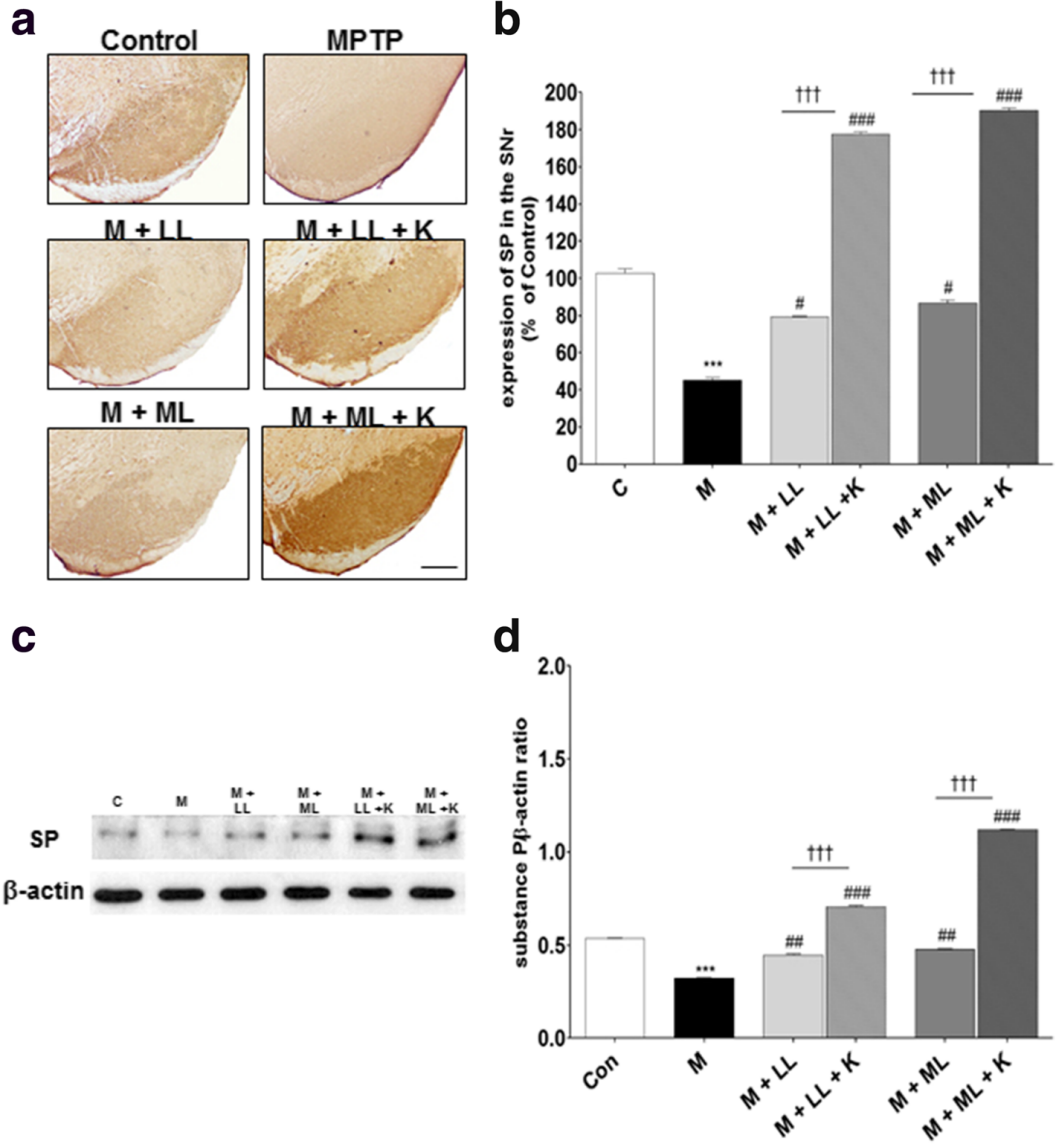
Effects of combination treatment on SP expression in the SNr. Immunohistochemical analyses of SP expression in the SNr (**a**, **b**). Western blot analyses of SP expression in the SNr (**c**, **d**). MPTP mice showed significantly lower SP expression in the SNr, whereas combination treatment significantly increased SP expression in the SNr. C: control, M: MPTP, K: KD5040, LL: low dose of _l_-dopa (5 mg/kg), ML: medium dose of _l_-dopa (10 mg/kg). *** *p* < 0.001 vs. control, ### *p* < 0.001 and # *p* < 0.05 vs. MPTP, and ††† *p* < 0.01 vs. each dose of _l_-dopa. Error bars represent SEM. Scale bar: 100 μm. SP: substance P; SNr: substantia nigra reticulate

During SP analysis, MPTP mice initially exhibited a higher expression of SP in the substantia nigra pars reticulata (SNr; 45.3 ± 6.3%, *p* < 0.001 vs. control) but later showed normalized levels after both doses of _l_-dopa treatment (LL: 79.4 ± 5.8%; ML: 86.8 ± 3.6%, both *p* values <0.05 vs. MPTP). Following combination treatment, there was a significantly higher expression of SP in both groups with LL (177.7 ± 12.5%, *p* < 0.001 vs. LL) and with ML (190.8 ± 9.0%, *p* < 0.001 vs. ML; Fig. 6a and b) groups.

Western blot analyses revealed similar trends as those of immunochemistry analyses. The expression of SP was lower in the MPTP group (60.5 ± 0.1%, *p* < 0.001 vs. control), but treatment with _l_-dopa alone (LL: 83.7 ± 0.1%; ML: 89.3 ± 0.1%, *p* < 0.01) and in combination with KD5040 (LL: 132.1 ± 0.1%; ML: 208.4 ± 0.1%, *p* < 0.001) resulted in a higher expression of SP. In particular, the combination treatment showed better synergistic effects (vs. each _l_-dopa group, *p* < 0.001; Fig. 5c and d, and Fig. 6c and d).

### Effects of combination treatment on glutamate content

**Fig. 7:**
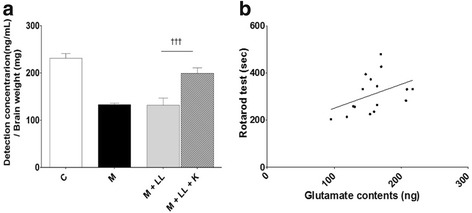
Effects of combination treatment on glutamate content in the motor cortex. Bar graph showing the glutamate content in motor cortex (**a**). Regression test between glutamate content and the rotarod test (**b**). A higher glutamate content had significant correlation with increased performance on the rotarod test. *** *p* < 0.001 vs. control group, ### *p* < 0.001 and # *p* < 0.05 vs. MPTP group, and ††† *p* < 0.001 and † *p* < 0.05 vs. each dose of _l_-dopa only. C: control, M: MPTP, K: KD5040, LL: low dose of _l_-dopa (5 mg/kg), ML: medium dose of _l_-dopa (10 mg/kg)

LC-MS/MS analyses revealed a lower glutamate content in the motor cortex of the MPTP group (133.0 ± 6.4 ng vs. 231.4 ± 9.5 ng [control], *p* < 0.001), and _l_-dopa alone (LL and ML) did not change these levels compared to the MPTP group (144.0 ± 8.8 and 149.3 ± 3.0 ng, respectively, both *p* > 0.05 vs. MPTP). However, after the combination treatment, glutamate content rose to 199.1 ± 3.3 ng with the LL (both *p* < 0.001 vs. MPTP and LL) and 164.1 ± 7.2 ng with the ML (both *p* < 0.05 vs. MPTP and ML; Fig. 7).

### Antidyskinetic effects of KD5040

**Fig. 8:**
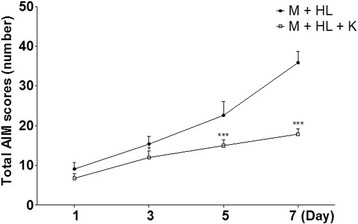
Effects of combination treatment on dyskinesia induced by _l_-dopa. AIMs were measured 20 min after the administration of _l_-dopa (20 mg/kg). M: MPTP, K: KD5040, HL: high dose of _l_-dopa (20 mg/kg). *** *p* < 0.001 and * *p* < 0.05 vs. MPTP + _l_-dopa (20 mg/kg). AIMs: abnormal involuntary movements

Next, to assess the antidyskinetic effects of KD5040, the AIM scores were assessed after _l_-dopa treatment (20 mg/kg, intraperitoneal [i.p.]). KD5040 improved the AIM scores when co-administered with a high dose of _l_-dopa (front paw: 6.75 ± 0.8 vs. 12.2 ± 1.7; three paw: 7.9 ± 1.8 vs. 16.0 ± 5.4; jumping: 3.2 ± 1.3 vs. 7.6 ± 1.4; all *p* values <0.001, vs. HL; Fig. 8). In addition, to confirm that the antidyskinetic effects induced by KD5040 were not due to a hypolocomotive effect, the cylinder wall touch and rotarod tests were performed. The HL group exhibited aggravated performance with peak dyskinesia, while combination treatment resulted in a significant reduction in impaired motor activity (data not shown).

### Striatal levels of FosB, pDARPP-32, pERK, and pCREB

**Fig. 9:**
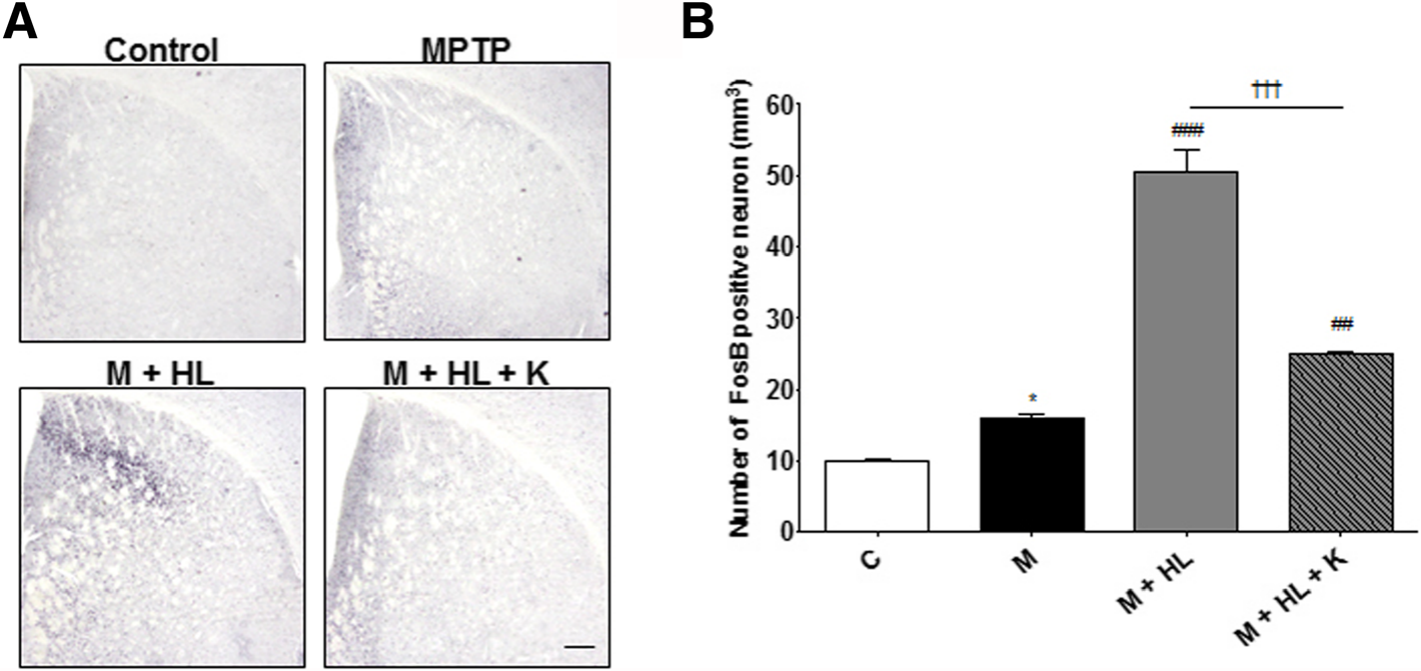
Effects of combination treatment on abnormal FosB activation in the striatum. Coronal sections showing FosB staining in each group (**a**). Graph showing altered FosB levels in the HL group compared to the control group and combination treatment group (**b**). C: control, M: MPTP, K: KD5040, HL: high dose of _l_-dopa (20 mg/kg). * *p* < 0.05 vs. control group, ### *p* < 0.001 and ## *p* < 0.01 vs. MPTP group, and ††† *p* < 0.001 vs. each dose of _l_-dopa. Scale bar: 200 μm

**Fig. 10:**
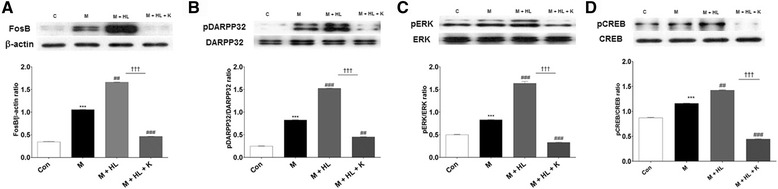
Effects of combination treatment on protein expression in the striatum. Representative Western blot images (upper panel) and quantifications (lower panel) of four proteins showing significant differences between groups. Protein analyses of FosB (**a**), pDARRPP32 (**b**), pERK (**c**) and pCREB (**d**). Western blot analyses showing significantly lower expression levels of FosB, pDARRPP32, pERK, and pCREB after combination treatment. C: control, M: MPTP, K: KD5040, HL: high dose of _l_-dopa (20 mg/kg). * *p* < 0.05 vs. control group, ### *p* < 0.001 and ## *p* < 0.01 vs. MPTP group, and ††† *p* < 0.001 vs. each dose of _l_-dopa

Immunohistochemical analyses revealed that HL alone induced higher striatal FosB levels (50.5 ± 6.4 cells/mm^3^, *p* < 0.001 vs. MPTP), whereas combination treatment alleviated this increase (25.0 ± 0.8 cells/mm^3^, *p* < 0.001 vs. HL; Fig. 9a and b). A correlation analysis showed that higher FosB levels were associated with LID (*r* = 0.9, *p* < 0.05); a Western blot analysis showed similar trends (Fig. 10a). In addition, the expression levels of pDARPP32, pERK, and pCREB were associated with KD5040-induced antidyskinetic effects. The combination treatment significantly lowered the expression levels of pDARPP-32, pERK, and pCREB (vs. HL, each *p* value <0.001; Fig. 10b–d), which were upregulated under HL challenge.

### Composition of KD5040

**Fig. 11:**
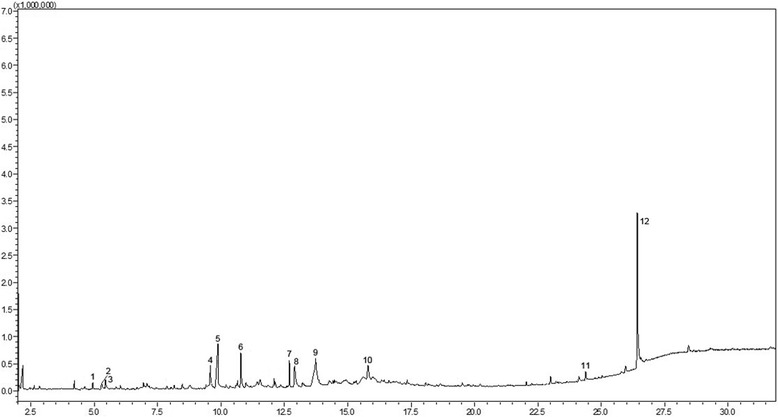
GC-MS analyses of KD5040. The X-axis represents retention time, and the Y-axis represents abundance

GC-MS analyses identified 12 major compounds in KD5040 (Fig. 11); their physicochemical and spectroscopic data are shown in Table 1.

**Table 1:** Quantification of compounds in KD5040 using gas chromatography-mass spectrometry

No.	Compound Name	RT (min)	Area
1	2-Furanmethanol	4.942	57,080
2	Ethanamine	5.291	157,960
3	1-Butanol, 2-amino-3-methyl	5.445	148,390
4	4H–Pyran-4-one, 2,3-dihydro-3,5-dihydroxy-6-methyl	9.576	121,071
5	Benzoic acid	9.875	816,509
6	5-Hydroxymethylfurfural	10.779	476,687
7	Eugenol	12.695	122,008
8	1,2,3-Benzenetriol	12.905	490,529
9	Adenosine, N6-phenylacetic acid	13.724	1,065,891
10	Quinic acid	15.801	213,470
11	Hexadecanoic acid	24.38	22,373
12	13-Docosenamide	26.419	851,211

## Discussion

The present study investigated the beneficial effects of combination therapy with _l_-dopa and KD5040 in a mouse model of PD induced by MPTP. KD5040 treatment enhanced the effectiveness of low dose _l_-dopa and the administration of KD5040 with HL significantly reduced LID.

_L_-dopa remains the gold standard pharmacological treatment for PD patients, but long-term administration of this drug leads to serious adverse effects called LID. Although a low dose is one way to ameliorate the adverse effects of _l_-dopa [12], treatment effectiveness may be greatly reduced [13]. To observe whether the combination treatment could compensate this limitation, the synergic effects of KD5040 with the minimum dose of _l_-dopa was tested. A medium dose of _l_-dopa (10 mg/kg) improved motor function, while a lower dose (5 mg/kg) alone did not reach therapeutic significance for improving motor symptoms, as expected. However, combination treatment with KD5040 and a low dose of _l_-dopa resulted in the improved motor function, which reached the level of a medium dose of _l_-dopa. Thus, the present results suggest that a combination treatment with KD5040 and _l_-dopa might reduce the effective dose of _l_-dopa needed to reach the therapeutic window (Figs. 2, 3 and 4).

**Fig. 12:**
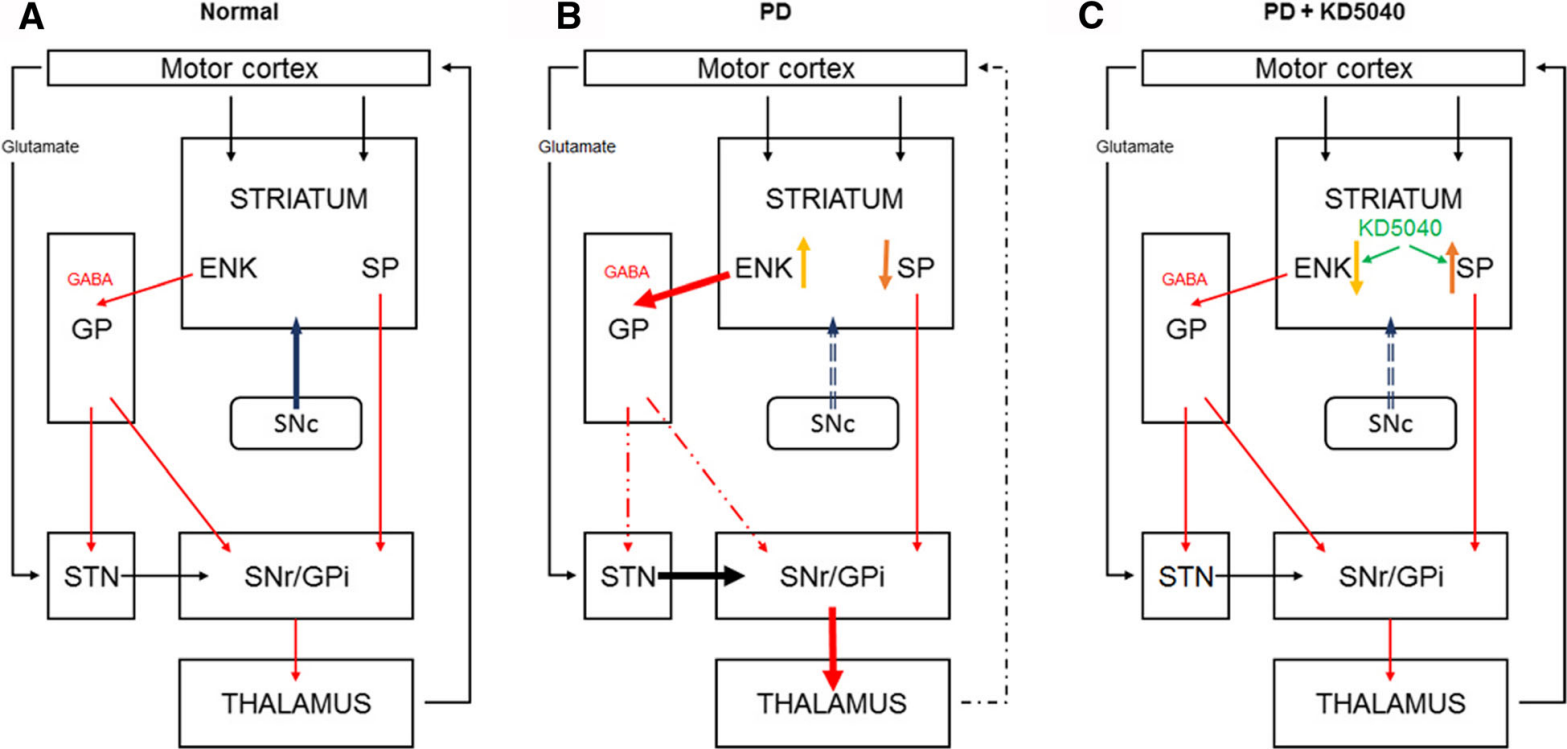
Basal ganglia motor circuits in normal (**a**), PD (**b**), and combination treatment (**c**) groups. Two families of receptors, D1 and D2, mediate the actions of dopamine in the basal ganglia. The progressive loss of dopaminergic neurons in the SNc causes PD and results in an imbalance of activities between the direct and indirect pathways. However, combination treatment enhanced motor function by correcting the imbalanced activity between the indirect and direct pathways in the basal ganglia. GP: globus pallidus, STN: subthalamic nucleus, GPi: internal globus pallidus, SNr: substantia nigra pars reticulate, SNc: substantia nigra pars compacta, PD: Parkinson’s disease

In PD patients, the inability to control voluntary movements is a consequence of organizational changes in basal ganglia circuits [2, 3]. The indirect pathway is innervated by ENK-containing GABA neurons within the GPe, whereas the direct pathway is innervated by SP-containing GABA neurons projecting to the internal GP and SNr [14–16]. A balanced working of these two pathways is important for the efficient control of movements [17, 18]. However, a depletion of dopamine due to PD increases activity within the indirect pathway and reduces activity within the direct pathway [17], resulting in a lack of control of basal ganglia outflow to the thalamus and inability to perform effective movements. To determine how the combination therapy synergistically improved motor function, the levels of key neuropeptides (SP and ENK) that play an important role in the modulation of dopaminergic pathways in the basal ganglia were assessed [19]. In accordance with the previous studies [19], altered levels of ENK and SP were observed in the MPTP-induced model of PD such that the ENK levels increased in the GPe and SP levels decreased in the SNr. However, the combination treatment reversed the MPTP-induced reduction in SP in the SNr as well as MPTP-induced increases in ENK in the GPe (Figs. 5 and 6), resulting in the imbalance between the indirect and direct pathways in the basal ganglia being rectified (Fig. 12). The degeneration of dopaminergic neurons in the SNpc is associated with aberrant glutamatergic innervation in the brain [20]; for example, changes in the glutamatergic input of the motor cortex have been observed in a parkinsonian state [21, 22]. In the present study, a lower glutamate content of the motor cortex in the MPTP group was observed, but the combination treatment helped restore the glutamate content (Fig. 7a). Moreover, motor function was positively associated with glutamate content in the motor cortex during the rotarod test (Fig. 7b).

**Fig. 13:**
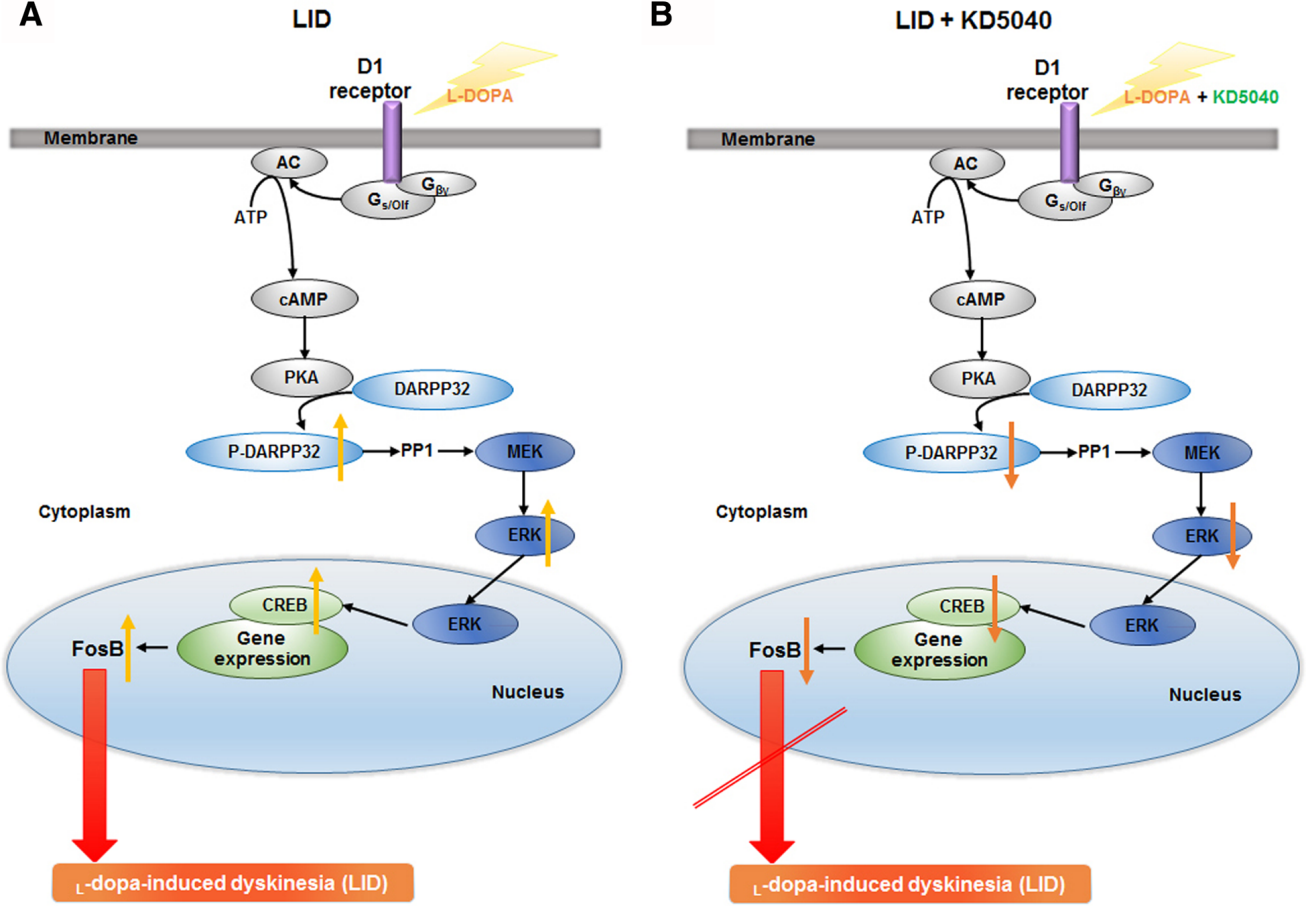
Effects induced by _l_-dopa in the striatal MSNs of the direct pathway. **a** MPTP plus a high dose of _L_-dopa (20 mg/kg) not only replaced dopamine in terms of excessive concentrations but also overactivated the D1 receptor to cause hyperactivity in the direct striatonigral pathway. **b** Combination treatment resulted in a significant normalization of the D1 pathway, resulting in antidyskinetic effects. MSNs: medium spiny neurons

PD is commonly treated using _L_-dopa, but its long-term use leads to several motor complications known as LID [23], seriously limiting the efficacy of current pharmacological treatments for PD [24]. In the present study, KD5040 had antidyskinetic actions on a mouse model of PD when used in combination with _l_-dopa. Recent studies have demonstrated that _l_-dopa activates D1 receptors (D1Rs), resulting in the overstimulation of the direct pathway [24]. The administration of _l_-dopa induces hyperactivation of cAMP-dependent protein kinase (PKA) and phosphorylation of DARPP32 at Thr34 in the dopamine-depleted striatum [25, 26]. The inhibition of DARPP32 in striatonigral neurons attenuates LID in a 6-OHDA-induced hemi-lesion model of PD [27]. ERK has also emerged as a key signaling component involved in the control of gene expression and synaptic plasticity [28], and the stimulation of the D1R/PKA/DARPP32 cascade leads to the phosphorylation and activation of ERK [29], which controls a variety of downstream effector proteins in the nucleus and cytoplasm [25, 28, 30]. In the nucleus, DARPP-32 and ERK signaling induces the phosphorylation of CREB, which in turn increases the expression of immediate early genes such as *fosB*. It was reported that higher level of ΔFosB in the medium spiny neurons are associated with LID via the activation of the D1R/cAMP cascade [31]. In the present study, the phosphorylation levels of DARPP32, ERK, and CREB were associated with a successful production of LID. However, interestingly, KD5040 could counteract with these activations (Fig. 10). Furthermore, HL increased striatal FosB expression in the striatum of dyskinetic mice while combination treatment markedly reduced them (Figs. 9 and 10a). And there was a correlation between LID scores and FosB expression levels (*p* < 0.05), which indicates that KD5040-induced antidyskinetic effects might be related to the shifts in the levels of FosB. Taken together, the present data suggest that KD5040 might regulate LID via the D1R/cAMP pathway (Fig. 13).

A GC-MS analysis of KD5040 revealed that this formulation consists of 12 major compounds (Fig. 11, Table 1). To better understand the role that KD5040 plays in the alleviation of PD symptoms, reference compounds from each herb need to be specified and quantified. For instance, eugenol, a compound identified in KD5040 by the present study, has been reported to have antioxidant, anti-inflammatory, and neurorestorative capabilities as well as the ability to inhibit amyloid formation [32–35]. In addition, one of the beneficial effects of KD5040 is that it may contain multiple active compounds that target multiple sites. This is similar to the concept of systems biology, which considers interactions among components in a network at various levels [36]. Therefore, studies aiming to elucidate the detailed mechanism(s) underlying the interactions among and the synergistic effects of these compounds will be necessary.

Many studies have focused on the development of agents to attenuate the degenerative processes associated with PD [37]. However, critics have argued that neuroprotective treatments cannot fulfill the therapeutic window for PD [38] because PD patients already exhibit more than 50–60% dopaminergic deficits in the nigrostriatal pathway when they are diagnosed as PD [39]. Nevertheless, the present findings indicate that KD5040 produces synergistic effects with _l_-dopa in terms of modulating basal ganglia circuits, in addition to the neuroprotective properties as shown in previous study [6]. Moreover, KD5040 can mitigate LID, which is a serious adverse effect due to the overdose or long-term use of _l_-dopa. However, the present results should be interpreted with caution because the effects of this combination treatment were tested in only one type of PD model. Thus, to confirm the translational value of KD5040, further studies using other induced and/or genetic models of PD will be necessary.

## Conclusions

Combination treatment with KD5040 and _l_-dopa lowered the effective dose of _l_-dopa and alleviated symptoms of LID, which is an adverse effect associated with overuse of _l_-dopa. Our findings suggest that KD5040 can be a possible candidate for adjunct therapy in treating motor dysfunction and dyskinesia in PD patients.

## Abbreviations

10 mg/kg, ML: A medium dose of _L_-dopa
20 mg/kg, HL: A hight dose of _L_-dopa
5 mg/kg, LL: A low dose of _L_-dopa
AIMs: Abnormal involuntary movements
CGT: Cheong-Gan-Tang
CREB: cAMP response element binding protein
D1Rs: D1 dopamine receptors
DARPP-32: Dopamine- and cAMP-regulated neuronal phosphoprotein
ECT: *Eugenia caryophyllata* Thunb
ENK: Enkephalin
ERK: Extracellular signal–regulated kinases
GC-MS: Gas chromatography-mass spectrometry
GP: Globus pallidus
GPe: Globus pallidus external segment
LC-MS/MS: Liquid chromatography tandem mass spectrometry
_L_-dopa: L-3, 4-dihydroxyphenylalanine
LID: _L_-dopa-induced dyskinesia
MPTP: 1-methyl-4-phenyl-1, 2, 3, 6-tetrahydropyridine
PCB: *Pogostemon cablin* Bentham
PD: Parkinson’s disease
SNpc: Substantia nigra pars compacta
SP: Substance P
